# Editorial: Cognitive and affective factors in relations to learning

**DOI:** 10.3389/fpsyg.2022.1037332

**Published:** 2022-09-29

**Authors:** Mikaela Nyroos, Johan Korhonen, Riikka Mononen

**Affiliations:** ^1^Department of Education, Umeå University, Umeå, Sweden; ^2^Faculty of Education and Welfare Studies, Åbo Akademi University, Vaasa, Finland; ^3^Teachers, Teaching, and Educational Communities, University of Oulu, Oulu, Finland; ^4^Department of Special Needs Education, University of Oslo, Oslo, Norway

**Keywords:** achievement, epistemic emotions, home learning environment, motivation, math anxiety, working memory

Both domain general (e.g., working memory, executive functions) and domain specific (e.g., number processing, phonological processing) cognitive factors have been found to predict learning in different age groups (e.g., Schneider et al., 2017; Peng et al., 2018). Likewise, research has shown that various affective factors such as different emotions (e.g., Camacho-Morles et al., 2021; Caviola et al., 2022) need to be considered when investigating individual differences in learning. However, less studies have investigated both cognitive and affective factors simultaneously in relation to learning. There is a lack of studies investigating the interplay (i.e., moderation and mediation) between cognitive and affective factors on learning.

The aim of this Research Topic is to deepen our knowledge on the cognitive and affective factors in relation to learning in different age groups. Providing a broad scope of emerging areas in research that simultaneously look at the interplay of these constructs related to academic learning, as well as longitudinally, the collection spans research methods and analyses, from innovative study designs to recent advances in methodology in this field. The volume comprises of two systematic reviews and 11 original research papers.

In their study, Koponen et al. show that educational interventions providing opportunities to practice and to perform successfully in math tasks, have positive effects on elementary school children's progress and change their belief about their own math skills, especially for children struggling with math. Thus, skill training math and teaching children to believe they can do and learn math is argued to enhance math development in general and specifically for low-performing children.

Zhang et al. combined the social cognitive career theory (SCCT) and the stimulus-organism-response (SOR) model and investigated university students' psychological cognition and attitudes in their learning. Their findings showed that self-efficacy and students' generic skills (e.g., self-management skills, learning and adaptability skills, problem-solving skills, concept, and analysis skills) had a mediating effect, when predicting learning satisfaction on either social support system or interaction relationship.

Moving to online learning, Wang Y. et al. conducted an online experimental study with 177 college students where they measured real-time emotions (joy and anxiety) with facial expressions and found that feelings of joy were positively related, and anxiety negatively related to students' self-reliance persistence. In other words, students that experienced joy were more persistently trying to solve the task while anxious students engaged in task-avoidance behavior.

The findings of Vanbecelaere et al. study increases our understanding on the dynamics of the home learning environment (HLE) and its impact on first grade children's cognitive and non-cognitive outcomes in math and reading. They showed a significant relation between parents' perceptions and their anxiety toward math and reading. A significant relation was also found between the parents' perceptions toward reading and the frequency of home reading activities, but not for math. Apart from socioeconomic status playing a role in children's digit comparison skills and math anxiety, no other HLE factors were found to have a relation to children's outcomes.

Shi and Qu article makes an important contribution concerning the mediating effect of overall positive mental state on cognitive ability and students' academic performance. Their study demonstrates that personality characteristics and psychology health play a partially mediating role between cognitive ability and English performance. The authors highlight the importance of environmental feedback to promote students' academic development and enhance their psychology health.

Jonsson et al. comparative study on the role of intrinsic cognitive motivation, analyses the effects of creative mathematical reasoning (CMR) and algorithmic reasoning (AR) on upper secondary students' conceptual understanding in math. Their study demonstrates that the CMR group outperformed the AR group. Need for cognition was a significant predictor of CMR's math performance, but not for the AR's. Further working memory capacity was a strong predictor, regardless of the group. From a school practice viewpoint, students should be offered time and struggle with constructing their own solution methods using CMR and supporting their conceptual understanding in math.

The two following reviews focus again on math anxiety. Finell et al. conducted a meta-analysis and investigated the link between math anxiety, working memory, and math performance. They found that (1) math anxiety was related to working memory, and that (2) working memory mediated the relation between math anxiety and performance. Their study lends support to the Attentional Control Theory as one possible mechanism underlying the math anxiety-performance relationship. In Balt et al. systematic review, math anxiety, and especially ways to reduce it, was investigated. Even if no clear picture could be found of what math anxiety interventions should look like for school-aged children, both mathematical intervention and cognitive-behavioral intervention approaches showed promising effects. Their conclusion calls for intervention research aiming to mitigate math anxiety among school-aged children.

The article by Wiklund-Hörnqvist et al. sheds light if retrieval practice in learning Swahili-Swedish word-pairs is influenced by individual differences in need for cognition. Using both behavioral and functional magnetic resonance imaging evidence, they concluded that retrieval practice is effective also for individuals with lower levels of need for cognition, in other words, those with low intrinsic motivation. This result thus supports the use of retrieval practice in schoolwork among upper-secondary school students.

Next, the contribution by Li et al. looks at the effect of class competition on academic achievement among primary school students, while considering their learning anxiety and engagement. The article illustrates how class competition was found to have a negative effect on academic achievement by increasing anxiety, but also a positive effect when promoting learning engagement. Thus, its role is important to acknowledge on academic achievement in relation to learning anxiety and engagement.

Dowker and Sheridan found math anxiety to be related to working memory, test anxiety, and math performance in a sample of 40 university undergraduate students. Female students experienced more math anxiety compared to males, but no gender differences were found in math performance. The authors argue that these findings lend support to the gender stereotype hypothesis postulating that gender differences in math emotions and motivation are mainly the result of students endorsing gender stereotypes like “boys are good at math”.

Vilhunen et al. examined epistemic emotions and their relation to learning. Curiosity and enjoyment were positively associated with learning, whereas frustration and boredom negatively. When controlling for pretest performance, only boredom showed negative relation to posttest performance. The authors discuss the complexity of interplay between emotions and learning, for example from the state vs. trait nature of the emotions.

Finally, Wang H. et al. examined the relations between the gender differences in boredom and lexicon learning in Chinese. They demonstrated that females experienced less boredom and their word forgetting rate was lower compared to males. This warrants for future studies, such as how feeling of boredom could be reduced in language learning situations.

In conclusion, we hope the present Research Topic will help to shed light on these new research perspectives. We also believe that these novel themes, combining cognitive and affective factors in relation to learning, together with their methodological approaches, may be of great value for professionals and practitioners.

## Author contributions

All authors listed have made a substantial, direct, and intellectual contribution to the work and approved it for publication.

## Conflict of interest

The authors declare that the research was conducted in the absence of any commercial or financial relationships that could be construed as a potential conflict of interest.

## Publisher's note

All claims expressed in this article are solely those of the authors and do not necessarily represent those of their affiliated organizations, or those of the publisher, the editors and the reviewers. Any product that may be evaluated in this article, or claim that may be made by its manufacturer, is not guaranteed or endorsed by the publisher.
